# Hipoplasia do Coração Esquerdo em Evolução até 21 Anos, Após Operação Cavopulmonar Total Realizada com 5 Anos de Idade

**DOI:** 10.36660/abc.20201012

**Published:** 2021-07-15

**Authors:** Edmar Atik

**Affiliations:** Instituto do Coração do Hospital das Clínicas Faculdade de Medicina Universidade de São Paulo São PauloSP Brasil Instituto do Coração do Hospital das Clínicas da Faculdade de Medicina da Universidade de São Paulo, São Paulo, SP - Brasil

**Keywords:** Síndrome do Coração Esquerdo Hipoplásico, Técnica de Fontan, Procedimentos Cirúrgicos Cardíacos

## Introdução

A hipoplasia do coração esquerdo caracteriza-se por dimensões muito reduzidas das cavidades cardíacas esquerdas, representadas pelo átrio esquerdo, valva mitral, ventrículo esquerdo, anel aórtico e aorta ascendente.^1^ A circulação sistêmica, assim, passa a ser dependente do canal arterial com fluxo reverso da artéria pulmonar hipertensa à aorta descendente e também à aorta ascendente. A circulação coronária também é dependente desse fluxo reverso, razão da grande chance de infarto do miocárdio, mesmo nos primeiros dias de vida. Torna-se fácil imaginar que, na diminuição da pressão arterial pulmonar ou do calibre do canal arterial, haja evolução desfavorável para baixo débito cardíaco. O coração direito se mostra dilatado e comunicação interatrial acompanha invariavelmente este defeito. Quando este pertuito se mostra de dimensões também reduzidas, conhecido como restritivo, ocasiona hipertensão pulmonar retrógrada. Por outro lado, quando de dimensões maiores pode favorecer a involução mais rápida da hipertensão pulmonar, com quadro congestivo e de baixo débito. Nos raros casos de septo interatrial íntegro, a drenagem anômala das veias pulmonares subsiste a fim da manutenção e viabilidade da circulação. Constitui-se a hipoplasia do coração esquerdo na quarta cardiopatia mais frequente no período neonatal, com alta mortalidade, ainda neste período, e corresponde a 1,5% de todas as cardiopatias congênitas.

**Como se exterioriza e evolui:** A instabilidade dinâmica favorece o advento de sinais e sintomas muito precoces na vida, nas primeiras horas ou dias de vida. Caracteriza-se por quadro congestivo (com comunicação interatrial grande) ou por baixo débito sistêmico (diminuição da pressão arterial pulmonar e/ou constrição do canal arterial), com surgimento de palidez, dispneia, desconforto, irritabilidade e cianose variável. O quadro mais favorável corresponde àquele com comunicação interatrial restritiva, que em vista da manutenção da hipertensão pulmonar, o débito cardíaco passa a ser mantido mais satisfatoriamente. Pulsos mais amplos nos membros inferiores e diminuídos nos superiores, ao lado de sopro sistólico discreto com segunda bulha hiperfonética na área pulmonar, sobrecarga de câmaras cardíacas direitas no eletrocardiograma e cardiomegalia decorrente das cavidades direitas, constituem-se em elementos diagnósticos. Na forma restritiva predomina a cianose, sem sopros e a congestão venocapilar pulmonar expressa a razão da hipertensão pulmonar retrógrada, responsável pela manutenção do fluxo sistêmico.

### Como se trata

**Clínica:** O uso até profilático da prostaglandina (0,05 a 0,1 mcg/Kg/min), logo após o nascimento, torna-se medida capital para a preservação do fluxo sistêmico através do canal arterial. Drogas vasoativas tipo dobutamina, dopamina e adrenalina se sobrepõem afim da manutenção da pressão pulmonar adequada, e também para manter o débito cardíaco.

Caso necessitem de intubação endotraqueal, é imprescindível que a fração inspirada de oxigênio não ultrapasse a 25% para evitar a vasodilatação pulmonar com consequente diminuição do débito cardíaco. Essas medidas se constituem em suporte para a obtenção da estabilização clínica (saturação de oxigênio em 80 a 85%) para evitar complicações pré-operatórias, de risco cirúrgico maior, como infarto do miocárdio, arritmias, disfunção ventricular, insuficiência tricúspide e baixo débito sistêmico.

**Cirurgia:** Duas técnicas principais são indicadas para a correção paliativa da hipoplasia do coração esquerdo. A mais empregada é a operação de Norwood clássica (transformação para anomalia tipo atresia pulmonar e dependente do Blalock-Taussig modificado) ou pela variante de Sano (transformação para anomalia tipo dupla via de saída de ventrículo direito com conexão por tubo sem valva, de 5 mm de diâmetro, entre o ventrículo direito e o tronco pulmonar). Em ambas técnicas, o tronco pulmonar é seccionado transversalmente, junto à bifurcação das artérias pulmonares e a ele se anastomosa a aorta ascendente, criando assim ampla conexão do ventrículo direito à aorta. Operação híbrida também pode ser realizada, com colocação de *stent* no canal arterial, bandagem das artérias pulmonares e ampliação da comunicação interatrial. A maior vantagem dessa técnica reside na eliminação da parada circulatória diminuindo o risco operatório nesta 1^a^ etapa. A indicação dessa técnica híbrida toma vulto principalmente em situações clínicas desfavoráveis em pacientes de alto risco, como na ausência de comunicação interatrial, em baixo peso corporal, no choque cardiogênico, em idades superiores a 14 dias e em centros ainda com alta mortalidade operatória.

**Como evolui após a operação:** O equilíbrio entre as resistências pulmonar e sistêmica deve ser mantido durante todo o tempo de pós-operatório. Esse aspecto é melhor obtido com a operação de Norwood-Sano, pois, nesta variante, não há desvio de sangue da circulação sistêmica através do Blalock-Taussig, a técnica clássica. Assim, o pós-operatório se torna mais estável, a perfusão coronária aumenta, assim como a esplâncnica e ainda diminui a sobrecarga de volume do ventrículo direito e a mortalidade, até a operação de Glenn. A desvantagem dessa técnica é a da ventriculotomia de cerca de 6 mm na via de saída ventricular que pode predispor à disfunção ventricular, arritmias e insuficiência pulmonar. Em relação à técnica híbrida, podem surgir problemas evolutivos, como a constrição do canal arterial, estenoses das artérias pulmonares em decorrência das bandagens prévias e até o desenvolvimento inadequado da aorta ascendente.

Na evolução de qualquer das técnicas preconizadas, teme-se o aparecimento de insuficiência cardíaca e da hipoxemia, mais pronunciadas. Daí a indicação precoce, cerca de 6 meses de idade para a 2^a^ etapa, pela operação de *Glenn* bidirecional e a 3^a^ etapa, para cerca de 18 a 24 meses de idade, a fim de se completar a operação cavopulmonar total.

A mortalidade total dos portadores de hipoplasia do coração esquerdo segundo a história natural, já no primeiro mês de vida, atualmente foi aliviada para cerca de 40 a 60% deles até a feitura do princípio Fontan.^2 - 5^ Em evolução posterior ao mesmo, a mortalidade também é alta, cerca de 25% deles em 10 anos e de até 55% em 30 anos.^2 , 3^

O propósito desta apresentação é mostrar uma boa evolução a longo prazo, superadas todas as fases operatórias preconizadas, e salientar as prerrogativas necessárias para essa boa evolução.

## Descrição do caso

### Dados clínicos

Paciente de 21 anos é acompanhado desde o nascimento com diagnóstico de hipoplasia do coração esquerdo, submetido à operação de Norwood com 4 dias de vida, técnica de Glenn bidirecional com 5 meses e cavopulmonar total com tubo externo não fenestrado com 5 anos. Desde então, evolui sem sintomas, em uso de warfarina, espironolactona e enalapril.

Exame físico: eupneico, acianótico, pulsos normais. Peso: 80 kg, Altura: 185 cm, PA: 110/80 mm Hg, FC: 65 bpm, O2 = 95%. Turgência jugular discreta e aorta discretamente palpada na fúrcula.

No precórdio, *ictus cordis* não era palpado e não havia impulsões sistólicas na borda esternal esquerda (BEE). As bulhas cardíacas eram normofonéticas e auscultava-se sopro sistólico discreto, +/++/4 de intensidade, timbre rude, na BEE baixa. O fígado não era palpado e pulmões limpos.

### Exames Complementares

**Figura 1:**
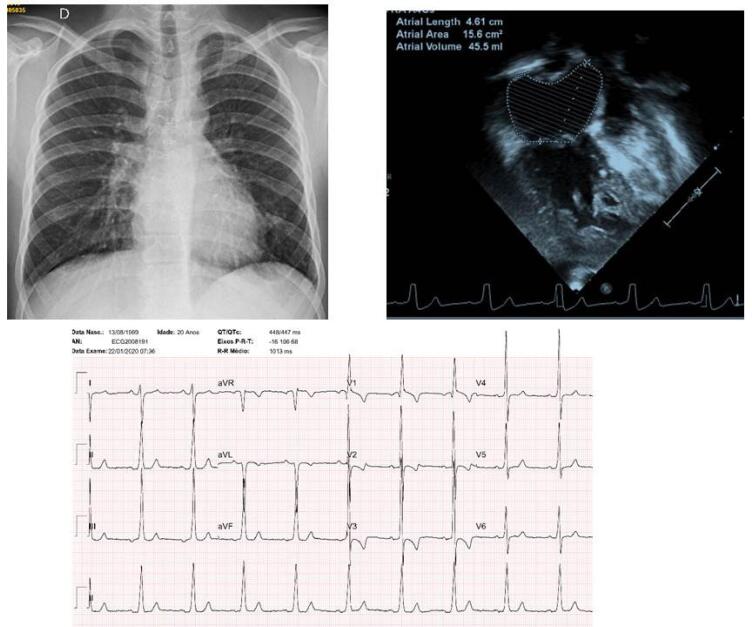
– Eletrocardiograma salienta a acentuada sobrecarga do ventrículo direito; a radiografia de tórax mostra área cardíaca e trama vascular pulmonar normais com discreto aumento da aorta; e o ecocardiograma em posição de 4 câmaras a hipoplasia acentuada da cavidade ventricular esquerda, estando normal o ventrículo direito sistêmico.

**Eletrocardiograma** mostrava ritmo sinusal e morfologia qR em V1 e onda T negativa de V1 a V3, indicativos de sobrecarga acentuada de ventrículo direito. Não havia potenciais de ventrículo esquerdo, com morfologia RS em V6. AP: +10^o^, AQRS: +100^o^, AT: +70^o^ ( Figura 1 ).

**Radiografia de tórax** mostra área cardíaca e trama vascular pulmonar normais. Havia discreta saliência do arco aórtico, e arco médio retificado ( Figura 1 ).

**Ecocardiograma** mostrou veia cava inferior dilatada (22 mm), tubo externo entre a veia cava inferior e a artéria pulmonar direita com fluxo laminar em velocidade de 0,68 m/s, anastomose da veia cava superior com a artéria pulmonar direita com velocidade máxima de 0,48 m/s. A conexão atrioventricular esquerda era ausente. Cavidade ventricular direita em conexão com a neoaorta (antiga valva pulmonar). Comunicação interatrial ampla com fluxo da esquerda para a direita. Ventrículo direito hipertrófico e dilatado com função sistólica preservada. Ventrículo esquerdo acentuadamente hipoplásico. Artérias pulmonares confluentes ( Figura 1 ).

**Tomografia de abdome** : Hepatoesplenomegalia com parênquima de densidade normal e homogêneo. Veias porta e hepáticas de calibre normal. Varizes esplênicas. Não há dilatação de vias biliares intra ou extra hepáticas. Demais estruturas abdominais sem alterações morfológicas.

**Diagnóstico clínico:** Síndrome de hipoplasia do coração esquerdo submetida à operação cavopulmonar total há 16 anos, em boa evolução a longo prazo.

### Características clínicas

**a. Raciocínio clínico:** Os elementos clínicos eram compatíveis com a boa evolução da operação de Fontan executada há 16 anos, em face de estar sem sintomas e em condição clínica favorável e sem intercorrências. O sopro sistólico auscultado na borda esternal esquerda não apresentava importância patológica por ser discreto, respaldado pela ausência de insuficiências valvares pelo ecocardiograma. A esperada sobrecarga acentuada do ventrículo direito, em face de ter se tornado o ventrículo sistêmico, não é acompanhada de disfunção, razão da preservação da boa evolução clínica do paciente.

**Conduta:** A evolução favorável clínica neste grupo de pacientes com hipoplasia do coração esquerdo decorre sempre da contínua preservação da função ventricular direita, mesmo submetida a uma carga pressórica maior, e por isso o uso da medicação vasodilatadora e em conformidade com a estrutura miocárdica. O anticoagulante necessário por alterações do fluxo sistêmico venoso lento acompanha todos os pacientes submetidos à operação de Fontan, a impedir a formação de trombos evolutivos neste sistema.

## Discussão

A hipoplasia do coração esquerdo, sem dúvida, continua sendo a anomalia congênita cardíaca mais temida, mesmo com a diminuição da mortalidade, verificada em todos os estágios evolutivos entre as operações necessariamente realizadas. Houve melhora da conduta imediata ao nascimento na preservação da condição clínica com débito sistêmico mais adequado, com o uso da prostaglandina E1 e de drogas vasoativas. Na sequência, o recém-nascido tem sido operado em melhor condição clínica e, assim, a técnica inicial de Norwood-Sano também se tornou de menor risco, e cerca de 90% tem sobrevivido nesta fase.^5^ As evoluções subsequentes, até a operação de Glenn e de Fontan, também se acompanham de menos intercorrências, o que em maior número conseguem ser acompanhados a maior prazo, como o paciente em questão. No entanto, apenas 40 a 50% desses pacientes alcançam a oportunidade da operação paliativa de Fontan.^2 , 5^ Assim, a morbidade de pacientes com hipoplasia do coração esquerdo continua elevada, indicando que mais cuidados devam ser tomados entre as fases operatórias.^2^ A evolução posterior à operação de Fontan obedece aos requisitos da técnica paliativa funcional com problemas relacionados a maior congestão venosa, ao menor débito cardíaco sistêmico, mas na preservação da boa função ventricular se torna mais favorável, como o observado. Em observação em 1052 pacientes com cardiopatias tipo ventrículo único, operados na Clínica Mayo entre 1973 e 2012, a sobrevida após 10, 20 e 30 anos da operação de Fontan foi de 74%, 61% e 43%. Problemas evolutivos se referem à pressão elevada no átrio esquerdo (>13 mm Hg), drenagem pleural prolongada (>21 dias), arritmias presentes, insuficiência renal, enteropatia perdedora de proteínas, disfunção cardíaca e reintervenções cirúrgicas nas valvas.

Sem dúvida, os desafios continuam, mas com sensíveis melhoras desde que Norwood mudou a história natural deste defeito em 1982.

## References

[1] . Atik E, Moreira VM. Hipoplasia do coração esquerdo. Atik E, Moreira VM, editors. Imagens e correlações em cardiologia pediátrica. São Paulo: Roca; 2011. p. 336-42.

[2] . Roeleveld PP, Axelrod DM, Klugman D, Jones MB, Chanani NK, Rossano JW, et al. Hypoplastic left heart syndrome: from fetus to fontan. Cardiol Young. 2018;28(11):1275-88. doi: 10.1017/S104795111800135X. 10.1017/S104795111800135X 30223915

[3] . Pundi KN, Johnson JN, Dearani JA, Pundi KN, Li Z, Hinck CA, et al. 40-year follow-up after the fontan operation: long-term outcomes of 1,052 patients. J Am Coll Cardiol. 2015;66(15):1700-10. doi: 10.1016/j.jacc.2015.07.065. 10.1016/j.jacc.2015.07.065 26449141

[4] . Khairy P, Fernandes SM, Mayer JEJ, Triedman JK, Walsh EP, Lock JE, et al. Long-term survival, modes of death, and predictors of mortality in patients with fontan surgery. Circulation. 2008;117(1):85-92. doi: 10.1161/CIRCULATIONAHA.107.738559. 10.1161/CIRCULATIONAHA.107.738559 18071068

[5] . Bautista-Hernandez V, Avila-Alvarez A, Marx GR, Del Nido PJ. Current surgical options and outcomes for newborns with hypoplastic left heart syndrome. An Pediatr. 2019;91(5):352.e1-352.e9. doi: 10.1016/j.anpedi.2019.09.007. 10.1016/j.anpedi.2019.09.007 31694800

