# Supplementary material for: Hipoplasia do Coração Esquerdo em Evolução até 21 Anos, Após Operação Cavopulmonar Total Realizada com 5 Anos de Idade
Source: Arq Bras Cardiol. 2021 Jul 15;117(1):142–5. [Article in Portuguese] doi: 10.36660/abc.20201012 (PMC8294718; doi:10.36660/abc.20201012)
Supplement: Translation

# Translation: Left Heart Hypoplasia Evolving Up to 21 Years, After Total Cavopulmonary Operation Performed at 5 Years of Age

**Authors:** Edmar Atik

**Affiliations:** Brazil Instituto do Coração do Hospital das Clínicas da Faculdade de Medicina da Universidade de São Paulo, São Paulo, SP - Brazil

**Keywords:** Hypoplastic Left Heart Syndrome, Fontan Procedure, Cardiac Surgical Procedures

## Introduction

Hypoplasia of the left heart is characterized by very small dimensions of the left cardiac cavities, represented by the left atrium, mitral valve, left ventricle, aortic ring and ascending aorta.^1^Systemic circulation thus becomes dependent on the ductus arteriosus with reverse flow from the hypertensive pulmonary artery to the descending aorta, and also to the ascending aorta. Coronary circulation is also dependent on this reverse flow, which is why there is a high chance of myocardial infarction, even in the first days of life. It is easy to imagine that, on decreased pulmonary arterial pressure or ductus arteriosus caliber, there is an unfavorable evolution towards low cardiac output. The right heart is enlarged and atrial septal defect invariably accompanies this defect. When this atrial defect appears to be of reduced dimensions, known as restrictive, it causes retrograde pulmonary hypertension. On the other hand, when larger, it may favor the faster involution of pulmonary hypertension, with congestive symptoms and low output. In the rare cases of intact atrial septum, anomalous pulmonary vein drainage remains in order of maintenance and viability of circulation. Left heart hypoplasia is the fourth most common heart disease in the neonatal period, with high mortality, even in this period, and corresponds to 1.5% of all congenital heart diseases.

**How it is externalized and evolves:** Dynamic instability favors the advent of very early signs and symptoms in life, in the first hours or days of life. These signs are characterized by congestive condition (with large atrial defect) or low systemic output (decreased pulmonary arterial pressure and/or constriction of the ductus arteriosus), with pallor, dyspnea, discomfort, irritability and variable cyanosis. The most favorable picture corresponds to the one with restrictive atrial septal defect, which, in view of the maintenance of pulmonary hypertension, the cardiac output is maintained at a satisfactory level.

Larger pulses in the lower limbs and decreased pulses in the upper limbs, alongside discrete systolic murmur with a second accentuated heart sound in the pulmonary area, overload of the right cardiac chambers on the electrocardiogram and cardiomegaly resulting from the right cavities, constitute diagnostic elements. In the restrictive form, cyanosis predominates, without murmurs and pulmonary venocapillary congestion expresses the reason for retrograde pulmonary hypertension, responsible for maintaining systemic flow.

### How it is Treated

**Clinic:** The prophylactic use of prostaglandin (0.05 to 0.1 mcg/kg/min), right after birth, is a key measure for the preservation of systemic flow through the ductus arteriosus. Vasoactive drugs such as dobutamine, dopamine and adrenaline are added together in order to maintain adequate pulmonary pressure, and to maintain cardiac output.

If they need endotracheal intubation, it is essential that the fraction of inspired oxygen does not exceed 25%, to avoid pulmonary vasodilation with a consequent decrease in cardiac output. These measures support clinical stabilization (oxygen saturation in 80 to 85%) to avoid preoperative complications, of greater surgical risk, such as myocardial infarction, arrhythmias, ventricular dysfunction, tricuspid regurgitation and low systemic output.

**Surgery:** Two main techniques are indicated for the palliative correction of left heart hypoplasia. The most used one is the classic Norwood operation (transformation to anomaly-type pulmonary atresia and dependent on the modified Blalock-Taussig technique) or by the Sano variant (transformation to anomaly-type double outlet right ventricle with non-valved tube connection, 5 mm in diameter, between the right ventricle and the pulmonary trunk). In both techniques, the pulmonary trunk is sectioned transversely, next to the bifurcation of the pulmonary arteries, and the ascending aorta is anastomosed to it, thus creating a wide connection between the right ventricle and the neoaorta. Hybrid operation can also be performed, with stent placement in the ductus arteriosus, banding of the pulmonary arteries and expansion of the atrial septal defect. The greatest advantage of this technique lies in the elimination of circulatory arrest, decreasing the operative risk in this first stage. Indication of this hybrid technique is particularly important in unfavorable clinical situations involving high-risk patients, such as in the absence of atrial septal defect, low body weight, cardiogenic shock, at ages older than 14 days and in centers with high operative mortality.

**How it evolves after the operation:** The balance between pulmonary and systemic resistance must be maintained throughout the postoperative period. This aspect is best achieved with the Norwood-Sano operation, since in this variant there is no diversion of blood from the systemic circulation through the classic Blalock-Taussig technique. Thus, the postoperative period becomes more stable, both coronary and splanchnic perfusion increase, and right ventricular volume overload and mortality decrease, until the Glenn operation. The disadvantage of this technique is a 6 mm ventriculotomy in the ventricular outflow tract that can predispose to ventricular dysfunction, arrhythmias and pulmonary insufficiency. Regarding the hybrid technique, evolutionary problems may arise, such as constriction of the ductus arteriosus, stenosis of the pulmonary arteries due to previous bandages and even the inadequate development of the ascending aorta.

In the evolution of any of the recommended techniques, heart failure and hypoxemia, which are more pronounced, are feared. Hence the early indication, about 6 months of age for the 2^nd^stage, by the bidirectional Glenn operation and the 3^rd^stage, for about 18 to 24 months of age, in order to complete the total cavopulmonary operation.

The total mortality of patients with hypoplasia of the left heart according to natural history, in the first month of life, has now been alleviated for about 40 to 60% of them until the completion of the Fontan^2 - 5^principle. In subsequent evolution, mortality is also high, about 25% of them in 10 years and up to 55% in 30 years.^2 , 3^

The purpose of this presentation is to show a good long-term evolution, overcoming all the recommended operative phases, and to highlight the necessary prerogatives for this good evolution.

## Case description

### Clinical data

A 21-year-old patient has been followed up since birth with diagnosis of left heart hypoplasia, who underwent Norwood operation at 4 days of life, 5-month bidirectional Glenn technique and total cavopulmonary anastomosis at 5 years with a non-fenestrated external tube. Since then, he has evolved without symptoms, using warfarin, spironolactone and enalapril.

Physical examination: eupneic, acyanotic, normal pulses. Weight: 80 kg, Height: 185 cm, BP: 110/80 mm Hg, CF: 65 bpm, O2 = 95%. Discreet jugular turgescence and aorta slightly palpated at the suprasternal notch.

In the precordium, apical impulse was not palpable and there were no systolic impulses at the left external border (LEB). The cardiac sounds were normal in intensity and a mild systolic murmur was heard, +/++/4 intensity, rough, in the low LEB. The liver was not palpable and the lungs were clean.

### Complementary Exams

**Electrocardiogram** showed sinus rhythm and qR morphology in V1 and negative T waves from V1 to V3, indicative of severe right ventricular overload. There were no left ventricular potentials, with RS morphology in V6. AP:+10^o^, AQRS: +100^o^, AT: +70^o^( Figure 1 ).

**Figure 1:** Electrocardiogram highlights the marked overload of the right ventricle; chest X-ray shows normal cardiac area and pulmonary vascular network with slight enlargement of the aorta; and the 4-chamber echocardiogram showed marked hypoplasia of the left ventricular cavity, being normal the systemic right ventricle.

**Chest radiography** shows normal cardiac area and pulmonary vascular network. There was a slight protrusion of the aortic arch, and a rectified medium arch ( Figure 1 ).

**Echocardiogram** showed dilated inferior vena cava (22 mm), external tube between the inferior vena cava and the right pulmonary artery with a laminar flow at a speed of 0.68 m/s, anastomosis of the superior vena cava with the right pulmonary artery with a maximum velocity of 0.48 m/s. The left atrioventricular connection was absent. Right ventricular cavity in connection with the neoaorta (former pulmonary trunk and valve). Wide interatrial communication with flow from left to right. Hypertrophic and dilated right ventricle with preserved systolic function. Left ventricle markedly hypoplastic. Confluent pulmonary arteries ( Figure 1 ).

**Abdominal tomography:** Hepatosplenomegaly with normal and homogeneous density parenchyma. Normal caliber hepatic and portal veins. Varicose splenic veins. No dilation of intra or extrahepatic bile ducts. Other abdominal structures without morphological abnormalities.

**Clinical diagnosis:** Left heart hypoplasia syndrome submitted to total cavopulmonary operation 16 years ago, in good long-term evolution.

### Clinical Characteristics

**a. Clinical reasoning:** The clinical elements were compatible with the good evolution of the Fontan operation performed 16 years ago, due to absence of symptoms and complications, and a favorable clinical condition. Systolic murmur auscultated on the left sternal border was not of pathological importance because it was discreet, supported by the absence of valve insufficiencies on echocardiogram. The expected marked right ventricular overload, as this has become the systemic ventricle, is not accompanied by dysfunction, which is why the patient’s good clinical evolution has been preserved.

**Management:** The favorable clinical evolution in this group of patients with hypoplasia of the left heart always results from continuous preservation of right ventricular function, even when subjected to a higher-pressure load and, for this reason, vasodilator medication was administered according to the myocardial structure. Anticoagulants required due to slow systemic venous flow abnormalities accompany all patients undergoing the Fontan operation, preventing the formation of evolutionary thrombi in this system.

## Discussion

Left heart hypoplasia is undoubtedly the most feared congenital cardiac anomaly, even with the decrease in mortality, as found in all evolutionary stages between the operations necessarily performed. There was an improvement in the immediate management at birth in preserving the clinical condition with more adequate systemic output, with the use of prostaglandin E1 and vasoactive drugs. As a result, the newborn has been operated in a better clinical condition, so the initial Norwood-Sano technique has also become less risky, and about 90% has survived in this phase.^5^Subsequent developments, up to the Glenn and Fontan operation, are also accompanied by fewer complications, which in greater numbers manage to be followed up in the long term, as the patient in question. However, only 40 to 50% of these patients have the chance of undergoing the Fontan palliative operation.^2 , 5^Thus, the morbidity of patients with hypoplasia of the left heart remains high, indicating that more care should be taken between the operative phases.^2^Evolution after the Fontan operation obeys the requirements of the functional palliative technique with problems related to greater venous congestion, lower systemic cardiac output but, in the preservation of good ventricular function, it turns out to be more favorable, as observed. Under observation, of 1052 patients with single ventricular type heart defects, operated on at the Mayo Clinic between 1973 and 2012, survival after 10, 20 and 30 years after the Fontan operation was 74%, 61% and 43%. Evolutionary problems refer to high left atrial pressure (>13 mm Hg), prolonged pleural drainage (>21 days), arrhythmias, renal failure, protein-losing enteropathy, cardiac dysfunction and surgical valve interventions.

Without a doubt, the challenges remain, but with noticeable improvements since Norwood changed the natural history of the left heart hypoplasia syndrome defects in 1982.
